# Anti-Nogo-A antibody treatment promotes recovery of manual dexterity after unilateral cervical lesion in adult primates – re-examination and extension of behavioral data

**DOI:** 10.1111/j.1460-9568.2009.06642.x

**Published:** 2009-03

**Authors:** Patrick Freund, Eric Schmidlin, Thierry Wannier, Jocelyne Bloch, Anis Mir, Martin E Schwab, Eric M Rouiller

**Affiliations:** 1 Unit of Physiology and Program in Neurosciences, Department of Medicine, Faculty of Sciences, University of Fribourg, Chemin du Musée 5CH-1700 Fribourg, Switzerland; 2 Brain Research Institute, University of Zürich and Department of Biology, ETH ZurichZürich, Switzerland; 3 Department of Neurosurgery, Neurosurgery Clinic, University Hospital of LausanneLausanne, Switzerland; 4 Neuroscience Research, Novartis Institute for BioMedical ResearchBasel, Switzerland

**Keywords:** hand, monkey, Nogo-A antibody therapy, spinal cord injury

## Abstract

In rodents and nonhuman primates subjected to spinal cord lesion, neutralizing the neurite growth inhibitor Nogo-A has been shown to promote regenerative axonal sprouting and functional recovery. The goal of the present report was to re-examine the data on the recovery of the primate manual dexterity using refined behavioral analyses and further statistical assessments, representing secondary outcome measures from the same manual dexterity test. Thirteen adult monkeys were studied; seven received an anti-Nogo-A antibody whereas a control antibody was infused into the other monkeys. Monkeys were trained to perform the modified Brinkman board task requiring opposition of index finger and thumb to grasp food pellets placed in vertically and horizontally oriented slots. Two parameters were quantified before and following spinal cord injury: (i) the standard ‘score’ as defined by the number of pellets retrieved within 30 s from the two types of slots; (ii) the newly introduced ‘contact time’ as defined by the duration of digit contact with the food pellet before successful retrieval. After lesion the hand was severely impaired in all monkeys; this was followed by progressive functional recovery. Remarkably, anti-Nogo-A antibody-treated monkeys recovered faster and significantly better than control antibody-treated monkeys, considering both the score for vertical and horizontal slots (Mann–Whitney test: *P* = 0.05 and 0.035, respectively) and the contact time (*P* = 0.008 and 0.005, respectively). Detailed analysis of the lesions excluded the possibility that this conclusion may have been caused by differences in lesion properties between the two groups of monkeys.

## Introduction

In the adult mammalian central nervous system, lesions lead to persistent motor and sensory deficits and the severity of these deficits is often correlated with the location and size of the injury. Transected nerve fibers do not spontaneously regrow in the central nervous system of adult mammals, due in part to the presence of myelin-associated neurite growth inhibitors such as Nogo-A (Filbin, 2003; Schwab, 2004; Yiu & He, 2006). After section of the corticospinal (CS) tract in adult rats, neutralizing Nogo-A with monoclonal antibodies leads to enhanced axonal regrowth and compensatory sprouting, in parallel with increased functional recovery (Schnell & Schwab, 1990; Bregman *et al.*, 1995; Thallmair *et al.*, 1998; Brösamle *et al.*, 2000; Schwab, 2004; Liebscher *et al.*, 2005).

Following incomplete spinal cord transection, in particular CS tract injuries, nonhuman primates show spontaneous functional recovery to a variable extent, depending on the lesion size and location, as well as on the difficulty of the behavioral task (Galea & Darian-Smith, 1997a,b; Sasaki *et al.*, 2004; Schmidlin *et al.*, 2004). Expanding upon data previously reported in rats, adult nonhuman primates were subjected to spinal cord injury and then treated with a neutralizing antibody against Nogo-A. They exhibited enhanced functional recovery, in parallel with increased sprouting in the CS tract, both caudal and rostral to the lesion site (in macaques: Freund *et al.*, 2006a, 2007; see also Fouad *et al.*, 2004 for anti-Nogo-A-enhanced regeneration of CS tract in marmosets subjected to spinal cord lesion). These studies thus emphasize the feasibility and importance of using adult nonhuman primate models of spinal cord injury as a precursor to clinical trials in humans (Lemon & Griffiths, 2005; Courtine *et al.*, 2007), especially considering the more similar organization of the CS system between nonhuman primates and humans (as compared to rats; see Nudo & Frost, 2006).

Some aspects of the study on anti-Nogo-A antibody-enhanced functional recovery and CS axonal sprouting in macaques following cervical cord lesion (Freund *et al.*, 2006a) were, however, questioned by two correspondence letters (Ho & Tessier-Lavigne, 2006; Tuszynski, 2006). Although we have responded to these criticisms (Freund *et al.*, 2006b), the aim of this report is to present additional data from the behavioral analysis and a more detailed statistical analysis. They clearly confirm that anti-Nogo-A antibody treatment indeed enhances the recovery of manual dexterity after cervical cord lesion (as compared to control antibody-treated monkeys). In particular, we have introduced a new parameter to measure manual dexterity, the ‘contact time’, which is defined as the time required to grasp the first food pellet targeted by the monkey. This new parameter evaluates manual dexterity more accurately as it assesses the precision grip between the thumb and the index finger, a prerogative of primates including humans (see Lemon & Griffiths, 2005; Lemon, 2008 for review). Finally, we have included measures to represent the estimated volume of the lesion in order to more comprehensively describe the cervical lesion.

## Materials and methods

**Fig. 1:**
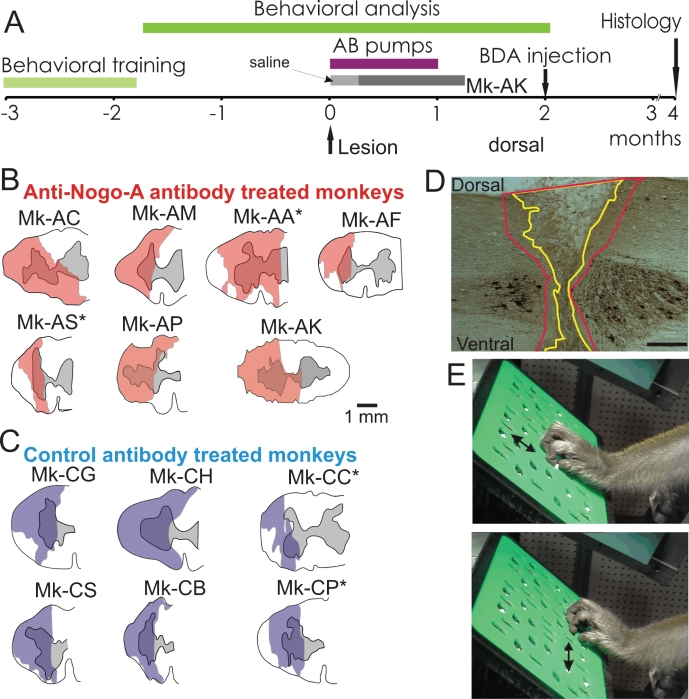
(A) Experimental time course, (B–D) lesion location and extent and (E) manual dexterity test. (A) Time course of experiment. AB pump, osmotic pump implanted to deliver the antibody (control or anti-Nogo-A), at the time of lesion in 12 monkeys, over 4 weeks (purple horizontal line). The anti-Nogo-A antibody infusion was delayed by 1 week in monkey Mk-AK (dark gray horizontal line), but saline was infused during the first week (light gray horizontal line). The neuroanatomical tracer BDA was injected in the contralesional motor cortex (Freund *et al.*, 2006a, 2007). (B and C) Reconstruction in the frontal plane, from sagittal sections, of lesion extent at cervical level C7/C8 in all monkeys. The grey area represents the grey matter of the cervical cord. Note that, due to the lesion, the hemi-cord on the side of the lesion has been distorted. In four monkeys (*), the dorsolateral funiculus was not completely transected (see Table 1; also Freund *et al.*, 2006a). The lesions of twelve monkeys (all except Mk-AK) have been illustrated in previous reports (Freund *et al.*, 2006a, 2007). (D) Photomicrograph of a sagittal section of cervical cord taken from monkey Mk-CH, illustrating the lesion site. Rostral is to the left. The yellow contour outlines the scar of the lesion and the penumbra zone is outlined in red. (E) Photographs illustrating the modified Brinkman board task used to assess manual dexterity pre- and post-lesion. The board comprises 50 slots each containing a food pellet, 25 oriented vertically and 25 horizontally. The monkey grasps the pellet in vertical slots by performing the precision grip (opposition of index finger and thumb) in a direction in the prolongation of the forearm (arrow in top panel). For the horizontal slots, the precision grip is oriented along a nearly horizontal axis (arrow in bottom panel). Scale bar in D, 0.5 mm.

The experiments were carried out on thirteen (3.5–6.9 years old) rhesus (*Macaca mulatta*) or cynomolgus (*Macaca fascicularis*) monkeys (male or female, 3.0–5.0 kg; see Table 1), in accordance to the Guide for Care and Use of Laboratory Animals (ISBN 0-309-05377-3; 1996) and approved by local veterinary authorities, including the ethical assessment by the local (cantonal) Survey Committee on Animal Experimentation and a final acceptance delivered by the Federal Veterinary Office (BVET, Bern, Switzerland). The monkeys were obtained from our own colony in our animal facility (*Macaca fascicularis*) or were purchased (*Macaca fascicularis* and *Macaca mulatta*) from a certified supplier (BioPrim; 31450 Baziège; France), with the authorization to import the animals delivered by the Federal Veterinary Office (BVET, Bern, Switzerland). Three recent reports (Wannier *et al.*, 2005; Freund *et al.*, 2006a, 2007) describe the behavioral task analyzed in the present report (‘modified Brinkman board’ task; see Fig. 1E and also http://www.unifr.ch/neuro/rouiller/motorcontcadre.htm), the surgical procedures (including transection of the CS tract in the cervical cord at C7/C8 level), the treatment with the anti-Nogo-A (*n* = 7) or control (*n* = 6) antibodies and the neuroanatomical investigations (including assessment of spinal lesion location and extent). The antibodies’ characteristics and penetration in the central nervous system have been reported elsewhere (Weinmann *et al.*, 2006; Freund *et al.*, 2007). As previously reported in detail (Schmidlin *et al.*, 2004, 2005; Wannier *et al.*, 2005; Freund *et al.*, 2006a, 2007).

**Table 1:** Results from cervical cord-lesioned monkeys included in the present study with identification code

							Anti-Nogo-A antibody						
	Control antibody						(11C7)				(hNogo)		
	Mk-CC	Mk-CP	Mk-CG	Mk-CS	Mk-CB	Mk-CH	Mk-AS	Mk-AF	Mk-AP	Mk-AA	Mk-AM	Mk-AC	Mk-AK°
Species	*Mul*.	*Fasc*.	*Fasc*.	*Mul*.	*Fasc*.	*Fasc*.	*Mul*.	*Mul*.	*Fasc*.	*Mul*.	*Fasc*.	*Fasc*.	*Fasc*.
‘Experimenter blind’	No	Yes	Yes	No	Yes	Yes	No	No	Yes	No	Yes	Yes	Yes
ICMS	Yes	–	–	Yes	–	–	–	Yes	–	–	–	–	–
Hemisection extent (%)	38	45	51	63	75	90	41	56	58	72	80	85	86
Functional recovery (%)													
Score (vert)	91	88	95	42	76	59	100	56	100	85	96	100	100
Score (horiz)	72	83	85	0	79	41	100	52	95	93	71	100	100
Contact time (vert)	100	50	77	36	38	22	93	57	89	100	78	84	100
Contact time (horiz)	59	54	100	0	29	32	100	72	100	86	90	100	100
Completeness of dlf section	No	No	Yes*	Yes	Yes	Yes	No	Yes	Yes	No	Yes	Yes	Yes
	–	(BDA)	(BDA)	–	(BDA)	(BDA)	(BDA)	(BDA)	(BDA)	(BDA)	(BDA)	(BDA)	(BDA)
Extent of lesion (%)													
Dorsal column	0	14	39	47	31	72	9	2	48	100	74	44	58
CS and RS territory	61	64	70	87	93	100	48	73	100	57	100	100	100
Ventral column	30	24	0	19	38	60	12	0	8	43	5	100	100
Volume of lesion (mm^3^)													
(scar in SMI-32 sections)	2.198	1.782	1.802	3.781	2.912	2.862	1.733	2.076	1.348	6.961	4.576	4.577	3.08

Antibodies: 11C7, mAb 11C7; hNogo, mAB hNogoA. *Mul*., *macaca mulatta*; *Fasc*., *macaca fascicularis*; ICMS, intracortical microstimulation; vert, vertical; horiz, horizontal; dlf, dorsolateral funiculus; RS, rubrospinal tract. At the time of the experiment, monkeys were assigned codes that did not allow experimenters to determine whether the animal was infused with the control or the anti-Nogo-A antibody. New names were assigned to the monkeys during the writing of the manuscript to improve its readability. Under ‘Experimenter blind’ procedure, ‘Yes’ refers to monkeys for which the experimenters testing the monkeys and measuring lesion extent did not know which antibody has been administered to the corresponding animal. Under ICMS, ‘Yes’ refers to the three monkeys subjected to extensive ICMS sessions in the primary motor cortex (M1), whose data have been reported previously (Schmidlin *et al.*, 2004, 2005). Functional recovery (expressed as a percentage of the two behavioral parameters score and contact time) was assessed here based on the modified Brinkman board task, by comparing the performance pre- and post-lesion, as explained in detail in the Materials and methods section for each of the two parameters. In the row ‘Completeness of dlf section’, ‘Yes’ and ‘No’ indicate whether the dlf was or was not completely transected unilaterally; ‘(BDA)’ indicates that completeness of the section of the dlf unilaterally was assessed based on the BDA labeling of the CS tract immediately above the lesion, in addition to the location and extent of the lesion itself. *In the monkey Mk-CG, a very small contingent of CS axons, labeled with BDA, escaped from the lesion ventrally. However, these few axons then ran ventrally in the white matter, without giving rise to collaterals into the area immediately caudal to the lesion, and continued their trajectory further below to low thoracic segments. As a consequence, these few preserved CS axons did not participate to the reconstruction of the CS tract in the segments immediately caudal to the lesion and therefore the lesion of the dorsolateral funiculus was considered as complete in this animal too. For completeness, the extent of the lesion (as a percentage) is given separately for three sub-territories of white matter in the lesioned hemi-cord. The three territories are: the dorsal column (from midline dorsally up to the dorsal rootlet entrance); the ventral column (from midline ventral to the ventral rootlet exit) and, laterally, the territory between the dorsal and ventral rootlets corresponding mainly to the territories occupied by the CS and RS tracts. A value of 100%, in the dorsal column for instance, means that 100% of the dorsal column sub-territory was lesioned in the corresponding monkey. For Mk-AK^(o)^ the anti-Nogo-A antibody treatment was delayed for 1 week. Nevertheless, an osmotic pump containing NaCl was implanted immediately after lesion in order to be consistent with the protocol of the other monkeys. The data presented here in the 12 leftmost columns and rows 1–5 and 10–13 have been reported earlier (Freund *et al.*, 2006a).

The present study includes the same twelve previously reported monkeys (Freund *et al.*, 2006a, 2007) and a thirteenth monkey (Mk-AK), on which the experiment was completed later, and in which anti-Nogo-A antibody infusion was initiated 7 days post-lesion (Fig. 1A), in contrast to immediate infusion the day of the lesion in the other six anti-Nogo-A antibody-treated monkeys. However, in this thirteenth monkey (Mk-AK), although an osmotic pump was implanted immediately after the lesion (as was the case for all the other monkeys), only saline (NaCl 0.9%) was delivered during the first week, and delayed administration of the anti-Nogo-A antibody started 1 week post-lesion. In all cases, the antibody was delivered for a period of 4 weeks.

The monkeys’ identification codes refer to individual monkeys (Table 1 in Freund *et al.*, 2006a) and comprise, for the sake of clarity, a ‘C’ or an ‘A’ in the fourth character position, indicating whether the monkey was control antibody-treated or anti-Nogo-A antibody-treated, respectively. However, during the course of the experiments the animals had different names from which the experimenter could not deduce which antibody was infused, at least for the monkeys in which the experimenter-blind procedure was applied (Table 1).

Monkeys were housed in our animal facilities in rooms of 12 m^3^, each usually containing 2–4 monkeys free to move in the room and to interact with each other. In the morning, before behavioral testing, the animal keeper placed the monkeys in temporary cages for subsequent transfer to the primate chair. The monkeys had free access to water and were not food-deprived. The rewards obtained during the behavioral tests represented the first daily access to food. After the tests, the monkeys received additional food (fruits and cereals). The dexterity of each hand was assessed in all lesioned monkeys with a finger prehension task, specifically our modified Brinkman board quantitative test (Fig. 1E; see also Rouiller *et al.*, 1998; Liu & Rouiller, 1999; Schmidlin *et al.*, 2004). The tests were conducted using a Perspex board (10 cm × 20 cm) containing 50 randomly distributed slots, each filled with a food pellet at the beginning of the test (home-made behavioral apparatus). Twenty-five slots were oriented horizontally and twenty-five vertically. The dimensions of the slots were 15 mm long, 8 mm wide and 6 mm deep. Retrieval of the food pellets required fractionated finger movements, in order to produce an opposition of the index finger and the thumb, which corresponds to the precision grip. This manual prehension dexterity task was executed daily, alternatively with one and the other hand, four or five times per week for several months before and after the unilateral cervical cord lesion. A daily behavioral session typically lasted 60 min. The performance of each hand was videotaped. In the present study, two parameters were assessed: (i) the retrieval score, i.e. the number of wells from which the food pellets were successfully retrieved and brought to the mouth during 30 s, separately for the vertical and the horizontal slots; (ii) the contact time, defined as the time of contact (in s) between the fingers and the pellet, calculated for the first vertical slot and the first horizontal slot targeted by the monkey in a given daily session (see also paragraph 2 in the Results section). The contact time is comparable to the prehension time as introduced by Nishimura *et al.* (2007) in parallel to the present study, but for a different grasping task. In our previous study, the retrieval score represented the primary outcome measure from the modified Brinkman board task (Freund *et al.*, 2006a) and was determined by the total number of pellets retrieved in 30 s. In the present study separate scores are provided for vertical and horizontal slots. The contact time data and the bivariate and trivariate statistical analyses (see below) represent secondary outcome measures from the modified Brinkman board task, newly introduced in the present report.

After the monkeys reached a level of performance corresponding to a plateau (usually after 30–60 days of initial training), we used the score from 30–50 daily sessions to establish a pre-lesion behavioral score for each monkey. A unilateral cervical cord lesion was performed in thirteen monkeys as follows. Intramuscular injection of ketamine (Ketalar® Parke-Davis, 5 mg/kg, i.m.) was delivered to induce anesthesia followed by an injection of atropine (i.m.; 0.05 mg/kg) to reduce bronchial secretions. In addition, before surgery, the animal was treated with the analgesic Carprofen (Rymadil®, 4 mg/kg, s.c.). A continuous perfusion (0.1 ml/min/kg) through an intravenous catheter placed in the femoral vein delivered a mixture of 1% propofol (Fresenius®) and a 4% glucose solution (1 volume of propofol and 2 volumes of glucose solution) to induce a deep and stable anesthesia. The animal's head was placed in a stereotaxic headholder, using ear bars covered at their tip with local anesthetic. The surgery was carried out under aseptic conditions, with continuous monitoring of the following parameters: heart rate, respiration rate, expired CO_2_, arterial O_2_ saturation and body temperature. In early experiments, an extra intravenous bolus of 0.5 mg of ketamine diluted in saline (0.9%) was added at potentially more painful steps of the surgical procedure (e.g. laminectomy) whereas, in later experiments, ketamine was added to the perfusion solution and delivered throughout surgery (0.0625 mg/min/kg). The animal recovered from anesthesia 15–30 min after the propofol perfusion was stopped, and was treated post-operatively with an antibiotic (Ampiciline 10%, 30 mg/kg, s.c.). Additional doses of Carprofen were given daily (pills of Rymadil mixed with food) for about 2 weeks after the surgery. Following the cervical cord lesion, the animal was kept alone in a separate cage for a couple of days in order to perform a careful survey of its condition. The details of surgical procedures and lesioning are available in previous reports (Schmidlin *et al.*, 2004, 2005; Wannier *et al.*, 2005; Freund *et al.*, 2006a, 2007).

**Fig. 3:**
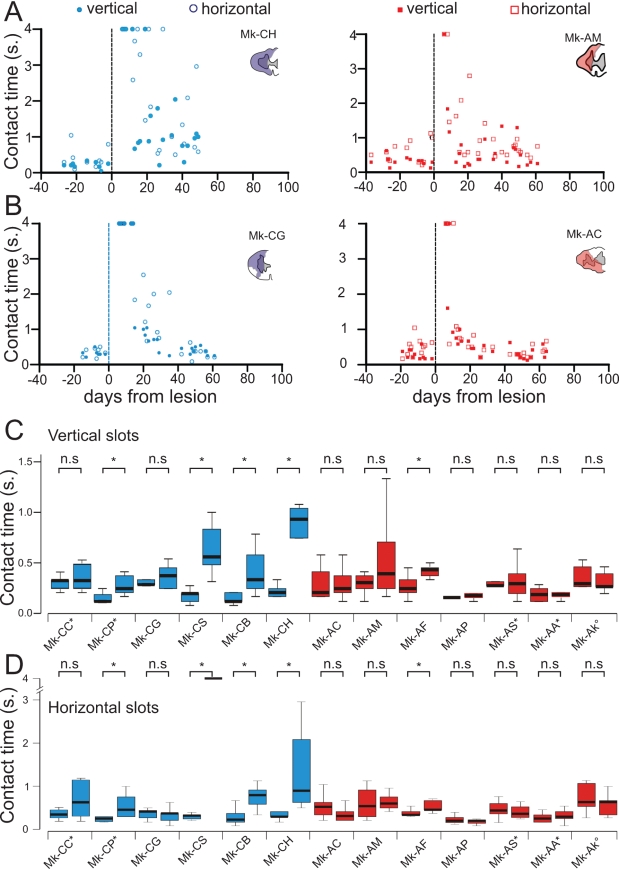
Quantitative assessment of manual dexterity based on the parameter contact time. (A and B) The contact time (in s), i.e. the time needed for one successful retrieval using the opposition of index finger and thumb in a pad-to-pad fashion, was plotted for two pairs of monkeys as a function of time (days) with respect to the lesion. The control antibody-treated monkeys are in the left column (blue) and the anti-Nogo-A antibody-treated monkeys in the right column (red). Data are shown before the lesion and over the post-lesion weeks for the vertically (filled symbols) and horizontally (open symbols) oriented slots. On the abscissa, day 0 (vertical dashed line) is for the time point of the cervical lesion. In rare cases shortly after the lesion, the contact time may have exceeded 4 s but was plotted here as 4 s. For each plot, the inset on the upper right is a reminder of lesion location and extent. (C and D) The contact times measured before lesion and within the last ten post-lesion sessions in the plateau reflecting stable recovery are distributed in form of box-and-whisker plots. For each monkey, the left box is for pre-lesion data whereas the right box is for post-lesion data. In box and whisker plots, the thick horizontal line in the box corresponds to the median value and the top and bottom of the box are for the 75th and 25th percentile values respectively. The top extremity of the whisker above the box is the largest data point included in the range going from the 75 percentile up to a level defined as the sum of the 75 percentile plus 1.5 times the inter-quartile distance. The bottom extremity of the whisker below the box is the smallest data point included in the range going from the 25 percentile down to a level defined as the 25 percentile minus 1.5 times the inter-quartile distance. *P <= 0.05 between pre- and post-lesion contact time values (Mann–Whitney test); n.s., nonsignificant difference (*P* > 0.05). Note in panel D that Mk-CS did not recover the ability to grasp the pellet post-lesion from horizontal slots and therefore the contact time was set to the upper time limit of the test, i.e. 4 s.

After lesion, and following the period of recovery lasting generally 30–40 days, a post-lesion level of performance corresponding to a plateau was established, based on a block of ten behavioral sessions (usually the last ten sessions conducted). For the retrieval score, functional recovery was expressed quantitatively as the ratio (expressed as a percentage) of the post-lesion average retrieval score value to the pre-lesion average score value. Because contact time was measured only for the first vertical and first horizontal slots targeted by the monkey, in order to minimize the impact of outliers the pre-lesion and post-lesion contact time was assessed as the median value (Fig. 3C and D). Considering that good performance is reflected by a short contact time (in the pre-lesion condition), post-lesion performance (recovery) was expressed quantitatively as the ratio (expressed as a percentage) of the pre-lesion median contact time to the post-lesion median contact time. For measures of both recovery of score and contact time, if the calculated values exceeded 100% (i.e. post-lesion performance was better than pre-lesion performance), the recovery was considered to be complete and therefore expressed quantitatively as 100%.

**Fig. 2:**
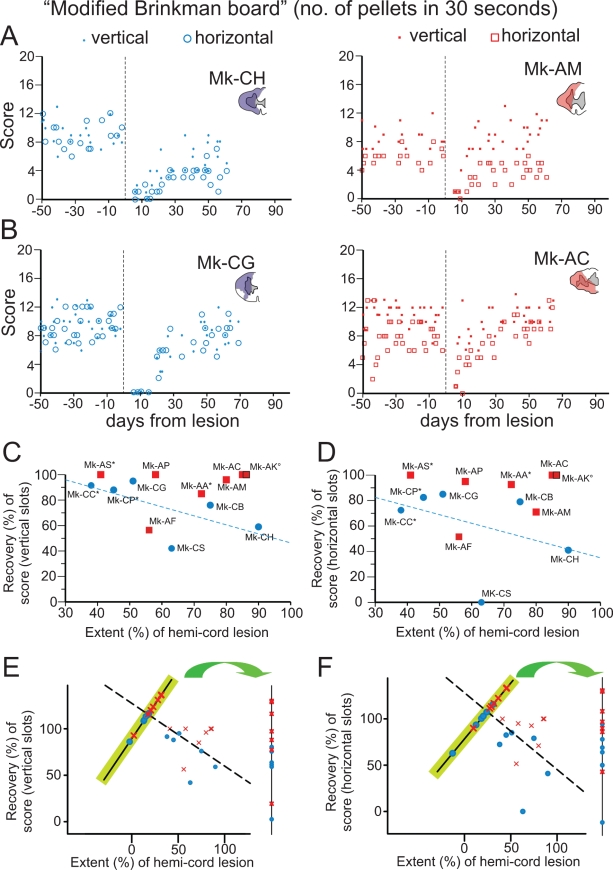
Quantitative assessment of manual dexterity based on the parameter score. (A and B) For two pairs of monkeys, comparison of retrieval scores obtained before and after the lesion (day 0, represented by the vertical dashed line) derived from the modified Brinkman board task (number of pellets retrieved in 30 s), for the hand affected by the cervical lesion. The control antibody-treated monkeys are in blue and the anti-Nogo-A antibody-treated monkeys in red. The scores were plotted separately for vertical (dots) and horizontal (open symbols) slots. For each plot, the inset is a reminder of lesion location and extent. (C and D) Relationship between the extent of hemi-cord lesion and degree of functional recovery of score (both as percentages) for the modified Brinkman board test for (C) vertically and (D) horizontally oriented slots (blue circles for control antibody-treated monkeys and red squares for anti-Nogo-A antibody-treated monkeys). The dotted line represents the tendency for an inverse correlation between recovery of score and lesion extent in the group of control antibody-treated monkeys (blue circles). In four monkeys (*), the dorsolateral funiculus was not completely transected (see Fig. 1B and C). The monkey Mk-AK differed from the others in that the post-lesion treatment was delayed by 1 week. (E and F) Illustration of the statistical analysis conducted on the data presented in C and D, respectively (for better visualization, circles are for control antibody-treated monkeys and crosses are for anti-Nogo-A antibody-treated monkeys). The dashed line has been computed such as to minimize the sum of distances between each data point and its projection onto the dashed line. Then, a solid line (in the green background rectangle) was drawn, orthogonal to the previously defined dashed line. Finally, the individual data points were projected on the solid line, along a direction parallel to the dashed line. In the green background zone the data points on the solid line become independent of the axes of the initial plot. The green arrow points to an enlargement of the solid line onto which the data points were projected (*n* = 6 for the control antibody-treated monkeys and *n* = 7 for the anti-Nogo-A antibody-treated monkeys). See Table 2 for corresponding statistics.

**Fig. 4:**
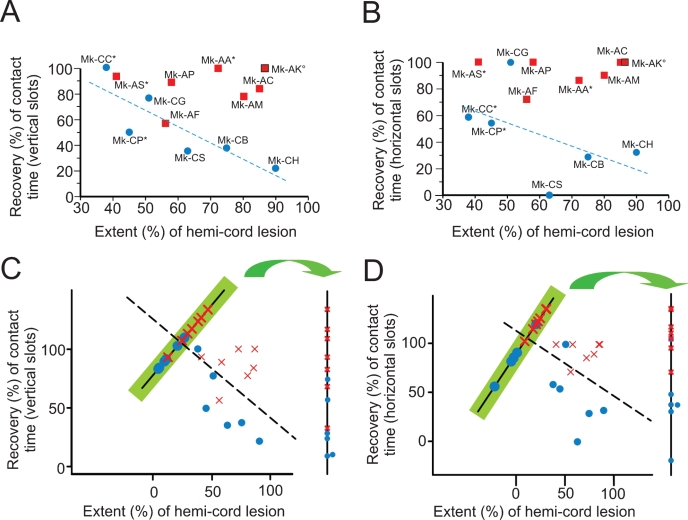
Relationship between the parameter contact time and hemi-cord lesion extent. (A and B) Relationship between extent of hemi-cord lesion and functional recovery for the contact time needed for the first successful picking. Same conventions as in Fig. 2C and D. The dotted line represents the tendency for an inverse correlation between recovery of contact time and lesion extent in the group of control antibody-treated monkeys (blue circles). (C and D) Graphic illustration of the statistical analysis conducted on the contact time data for (C) the vertical and (D) the horizontal slots. Same conventions as in Fig. 2E and F. See Table 2 for corresponding statistics.

Besides the new behavioral parameter of contact time introduced here, the present study also comprises a new analysis regarding the lesion size. In our previous reports (Freund *et al.*, 2006a, 2007), the extent of the lesion was expressed as a percentage of the corresponding hemi-cord surface, as assessed from a 2-D reconstruction of the lesion in the transverse plane (see Fig. 1B and C). These standard values of lesion extent have been considered here again in Figs 2 and 4 (see also Table 1). The present study expands upon these data by further calculating the estimated volume of the cervical lesion in order to consider the extent of the lesion in 3-D. After completion of the post-lesion behavioral analysis (see below), the monkeys were killed and prepared for histology as follows. Each monkey was pre-anaesthetized with ketamine (5 mg/kg, i.m.) and given an overdose of sodium pentobarbital (Vetanarcol; 90 mg/kg, i.p.). Transcardiac perfusion of saline (0.9%) was followed by paraformaldehyde (4% in phosphate buffer 0.1 m, pH 7.4), and 10, 20 and 30% solutions of sucrose in phosphate buffer. The brain and the spinal cord were dissected and stored overnight in a solution of 30% sucrose in phosphate buffer. Frozen sections (50 μm thick) of the cervical cord (approximately segments C6-T3) were cut in the parasagittal longitudinal plane and collected in three series for later histological processing (see below).

**Table 2:** *P*-values (Mann–Whitney test) for the statistical assessment of manual dexterity, comparing anti-Nogo-A antibody-treated monkeys (*n* = 7) and control antibody-treated monkeys (*n* = 6)

	*P*-values					
	Score (no. of pellets in 30 s)		Contact time		Lesion size	
	Vertical	Horizontal	Vertical	Horizontal	Vertical	Horizontal
Lesion extent (% of hemi-cord)						
(A) Bivariate analysis	0.05	0.035	0.008	0.005	–	–
(B) Trivariate analysis	–	–	–	–	0.072 (n.s.)	0.044
Estimated lesion volume (scar in SMI-32 sections)						
(C) Bivariate analysis	0.035	0.022	0.035	0.008	–	–
(D) Trivariate analysis	–	–	–	–	0.146 (n.s.)	0.031

In A, (bivariate analysis on the extent of the lesion and the percentage of functional recovery), *P*-values are given for the recovery of score (see Fig. 2E and F; vertical and horizontal slots, respectively) and recovery of contact time (see Fig. 4C and 4D; vertical and horizontal slots, respectively). In B, a trivariate rank test was conducted on the three parameters recovery of scores, recovery of contact time and extent of lesion, as described in Materials and methods. The same two statistical analyses used to treat the data in A and B were conducted on the data presented in C and D, but considering the estimated volume of the cervical lesion expressed in mm^3^ instead of its extent as a percentage of hemi-cord. In C and D, similar statistical conclusions (*P*-values < 0.05) were obtained when taking as volume of the lesion the values determined either from the lesion penumbra on SMI-32-stained sections or from the lesion as seen on BDA-stained sections. The size of the cervical lesion is given by its extent expressed as a percentage of hemi-cord (A and B) or by its estimated volume in mm^3^ (C and D).

**Fig. 5:**
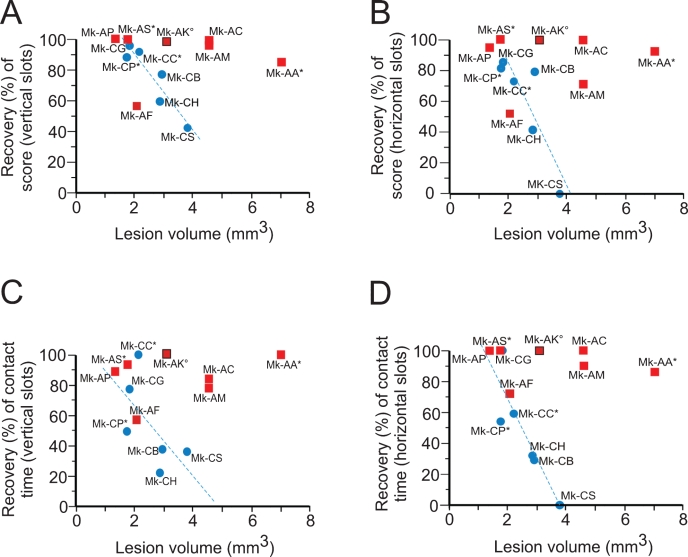
Relationship between the behavioural parameters score and contact time as a function of the estimated volume of the cervical lesion. (A and B) Relationship between the degree of functional recovery of score (as a percentage) for the modified Brinkman board test and the estimated volume of the cervical lesion for (A) vertically and (B) horizontally oriented slots (blue circles for control antibody-treated monkeys and red squares for anti-Nogo-A antibody-treated monkeys). (C and D) Relationship between the degree of functional recovery of contact time needed for the first successful retrieval and the estimated volume of the cervical lesion, for (C) vertically and (D) horizontally oriented slots (blue circles for control antibody-treated monkeys and red squares for anti-Nogo-A antibody-treated monkeys). In each panel, the dotted line represents the tendency for an inverse correlation between recovery of score or contact time and the estimated volume of the lesion in the group of control antibody-treated monkeys (blue circles). See Table 2 for corresponding statistics. In four monkeys (*), the dorsolateral funiculus was not completely transected (see Fig. 1B and C). The monkey Mk-AK differs from the others because post-lesion treatment was delayed by 1 week.

Using an *ad hoc* function of the Neurolucida software (based on the Cavalieri method; MicroBrightField, Inc., Colchester, VT, USA), the volume of the cervical lesion (in mm^3^) was extrapolated from the reconstructions of the lesion on consecutive histological longitudinal sections of the cervical cord (see Table 1). The volume measurement of the cervical lesion was conducted on one out of three series of sagittal sections (50 μm thick), treated immunocytochemically with the SMI-32 antibody (Covance, Berkeley, CA, USA), as previously reported (Liu *et al.*, 2002; Beaud *et al.*, 2008; Wannier-Morino *et al.*, 2008). The epitope recognized by the SMI-32 antibody lies on nonphosphorylated regions of neurofilament protein and is only expressed by specific categories of neurons (Campbell & Morrison, 1989; Tsang et al., 2006). The other two series of sections were processed to visualize biotinylated dextran amine (BDA; Invitrogen, Molecular Probe, Eugene, OR, USA) and fluorescein dextran amine (Invitrogen, Molecular Probe, Eugene, OR, USA) staining, resulting from injections of BDA in the contralesional motor cortex and fluorescein dextran amine in the ipsilesional motor cortex (see Freund et al., 2006a, 2007). Measurements of volume of the cervical lesion were also conducted on sections processed for BDA but the lesion contour was not as well defined as on the SMI-32-stained sections, where a clear scar region could be distinguished from a penumbra lesion at the periphery of the lesion (yellow and red outlines in Fig. 1D). The scar region was characterized by a dense fibrous tissue or granulous tissue forming a central zone of the lesion where the SMI-32 staining was absent. The lesion volume data presented (Table 1, Fig. 5) and considered for statistical analysis (Table 2) are the measurements corresponding to the scar as seen on the SMI-32-stained sections.

Because of the limited number of animals, two independent statistical tests were used to compare the group of control antibody-treated monkeys (*n* = 6) with the group of anti-Nogo-A antibody-treated monkeys (*n* = 7). The first test (based on a linear Fisher discriminant analysis) takes into account one of the two parameters reflecting the size of the lesion (i.e. the extent of hemi-cord lesion or the volume of the lesion) and one of the four parameters reflecting the percentage of functional recovery (score for vertical slots, score for horizontal slots; contact time for vertical slots or contact time for horizontal slots), and thus is aimed at assessing the overlap or segregation between the two groups of data (Figs 2E and F, and 4C and D). The test provides maximal separation between the groups (see Everitt, 2005) in the form of a linear function of the observed variables such that the ratio of the between-groups variance to its within-group variance is maximized. We used the *R* package to get the two lines plotted in each of Figs 2E and F, and 4C and D. Line 1 (dashed line) yields maximal separation and the projected samples are provided on the orthogonal line 2 (solid line). For better visualization, line 2 was proportionally enlarged and positioned vertically on the right side of the graph (green arrows). With respect to the statistics, the sample size does not permit an assumption of normality so we considered the statistical problem of separation of the projected samples using the nonparametric Mann–Whitney *U*-test. The obtained results are summarized in Table 2 (row A, bivariate analysis).

The second statistical test (the trivariate analysis) examined the three-dimensional data produced by differences in ‘recovery of scores’ (number of pellets retrieved, as illustrated in Fig. 2), ‘recovery of contact time’ (time to grasp first pellet, as illustrated in Fig. 4) and ‘lesion extent’, using a nonparametric multivariate rank test (Oja & Randles, 2004). This test includes all three parameters and can be considered an index of overall functional recovery. We assumed two independent random samples from bivariate distributions F(x-c1) and F(x-c2) located at centers c1 and c2, and tested the null hypothesis that there was no effect of treatment (i.e. c1 = c2 versus the alternative c1 is different from c2). Data were transformed to make the test affine-invariant, to ensure a consistent performance over all possible choices of coordinate system, and then projected onto a sphere where a rank test was performed. As the law for this test is still unknown, we used Monte-Carlo simulations to compute the *P*-value. The obtained results are summarized in Table 2 (row B, trivariate analysis). A complete description of these bivariate and trivariate statistical analyses, applicable also to other types of lesions and to other behavioral tests of manual dexterity in primates, will be reported elsewhere in a methodological report. The same two statistical analyses (bivariate and trivariate tests) were applied in a similar way as above for the estimated volume of the lesion (Table 2, rows C and D).

## Results

The experimental protocol is schematized in Fig. 1A. The location and extent of the cervical cord lesion is represented for all monkeys in Fig. 1B and C, including four animals in which the CS tract was not completely transected (asterisks). A typical lesion is shown in Fig. 1D. In three of thirteen monkeys (Mk-CS, Mk-CC and Mk-AF), an intracortical microstimulation mapping of the M1 hand area was conducted on both hemispheres (Schmidlin *et al.*, 2004, 2005), a procedure which may have partly reduced the recovery of their manual dexterity.

### Assessment of post-lesion recovery of manual dexterity: retrieval scores

Following unilateral cervical injury, the dexterity of the ipsilesional hand was severely impaired when assessed with the modified Brinkman board test. In our previous report (Freund *et al.*, 2006a) only the total retrieval score was presented whereas here, scores (number of pellets retrieved within the first 30 s) are presented separately for the vertical and horizontal slots (Fig. 2). Retrieval from horizontal slots is expected to be more difficult as it requires a postural adaptation of the hand in addition to the precision grip. Indeed, even when the control of the hand muscles is affected by the lesion, a hand posture naturally similar to that normally used for grasping pellets from the vertical slots is maintained (Fig. 1E). In contrast, the horizontal slots require the monkey to actively position the hand such that the axis between the extremities of the thumb and the index finger is oriented horizontally (Fig. 1E). For instance, when performing the precision grip with the right hand in the horizontal slots located on the left half of the modified Brinkman board, the index finger must be placed on the left and the thumb on the right (corresponding to a pronation and abduction of the wrist); in contrast, when aiming for the horizontal slots located on the right half of the modified Brinkman board, the index finger must be positioned to the right of the thumb (corresponding to a supination and adduction of the wrist).

For two monkeys tested in parallel by the same experimenter and having similar lesions (Fig. 2A), the anti-Nogo-A antibody-treated monkey (Mk-AM) exhibited faster and better recovery (96% and 71% of pre-lesion scores for the vertical and horizontal slots, respectively) than the control antibody-treated monkey (Mk-CH: 59% and 41%, respectively). Similarly (Fig. 2B), for two other monkeys tested in parallel, even though the anti-Nogo-A antibody-treated monkey (Mk-AC) had a larger lesion, this monkey recovered faster and slightly better (100% for both slot orientations) than the control antibody-treated monkey (Mk-CG: 95% and 85% for the vertical and horizontal slots, respectively).

The relationship between lesion extent and recovery of manual dexterity (i.e. score) in all monkeys, for vertically (Fig. 2C) and horizontally (Fig. 2D) oriented slots, shows that control antibody-treated monkeys (blue circles) were characterized by a tendency to an inverse correlation between lesion extent and percentage of recovery: *r* = −0.632 for the vertical slots (*P* = 0.178) and *r* = −0.395 for the horizontal slots (*P* = 0.438). In contrast, in most anti-Nogo-A antibody-treated monkeys (red squares), the recovery was substantially enhanced and, most importantly, reached 90–100% irrespective of the lesion extent (Fig. 2C and D): *r*=0.261 for the vertical slots (*P* = 0.571) and *r*=0.144 for the horizontal slots (*P* = 0.758).

In our previous report (Freund *et al.*, 2006a), the statistical analysis between the two groups of monkeys (anti-Nogo-A antibody- and control antibody-treated monkeys) was based on a Mann–Whitney test taking into consideration only the retrieval score, but not the lesion extent, a crucial parameter in the present matter, especially for the control antibody-treated monkeys (Fig. 2C and D). Thus, a new statistical procedure was introduced here to consider both the retrieval score and the lesion extent by plotting the data as in Fig. 2C and D but computing a line defined by minimizing the sum of distances between each data point and its projection onto the line (dashed lines in Fig. 2E and F). Then, data points were projected in the direction given by this line onto an orthogonal line segment (green background zone where the data points are independent of the axes of the initial plot). Finally, the overlap between the two treatment groups of monkeys (blue circles for control antibody-treated monkeys and red crosses for anti-Nogo-A antibody-treated monkeys) was assessed using the Mann–Whitney test. The anti-Nogo-A antibody-treated monkeys demonstrated significantly more recovery (as assessed by retrieval score) than the control antibody-treated monkeys, when tested for the vertical slots (*P* = 0.05) and for the horizontal slots (*P* = 0.035); see also,Table 2, row A.

### Assessment of post-lesion recovery of manual dexterity: contact time

The above data, based on the primary analysis of the modified Brinkman board task (score given by the total number of pellets retrieved in 30 s), reflect not only grasping ability but also other components of the movements such as the reaching phase, the variable time to chew the reward before aiming for the next pellet, etc, thus introducing more variability to the measure. In order to investigate solely the capacity of fine finger movement (i.e. the precision grip between the index finger and thumb), the grasping phase was analyzed by measuring the contact time from the video sequences. Contact time is defined as the time interval separating the first contact of the index finger with the food pellet and the final successful grasp of the pellet utilizing pad-to-pad opposition of the index finger and thumb (i.e. when the pellet leaves the slot), for, respectively, the first vertical and the first horizontal slots targeted by the monkey’s hand.

For the same two pairs of monkeys as in Fig. 2, the contact time was plotted as a function of time (in days) pre- and post-lesion (Fig. 3A and B). Before lesion, all monkeys exhibited a short and relatively stable contact time. Immediately following the lesion, contact time increased substantially and became more variable from one daily session to the next. However, the anti-Nogo-A antibody-treated monkeys (red symbols in Fig. 3A and B) recovered faster and better than the control antibody-treated monkeys (blue symbols in Fig. 3A and B).

The contact time in the vertical and horizontal slots has been plotted for all thirteen monkeys (box-and-whisker plots in Fig. 3C and D), showing the pre-lesion values adjacent to the post-lesion values after recovery. In four of the six control antibody-treated monkeys, for both the vertical and the horizontal slots, contact time values were significantly longer post-lesion than pre-lesion (Mann–Whitney, *P* < 0.05; Fig. 3C and D), corresponding to an incomplete recovery. In contrast, the post-lesion contact time values of six of the seven anti-Nogo-A-treated monkeys were not statistically significantly different (Mann–Whitney, *P* > 0.05) from pre-lesion values (Fig. 3C and D), thus reflecting substantial functional recovery.

Contact time values, expressed as percentage recovery, were plotted as a function of lesion extent for the vertical and horizontal slots, respectively (Fig. 4A and B). The percentage recovery of contact time was inversely correlated with the lesion extent in the control antibody-treated monkeys for the vertical slots with *r* = −0.840 (*P* = 0.036) whereas there was only a trend for the horizontal slots (*r* = −0.511; *P* = 0.301). In contrast, such a trend for an inverse correlation was absent in the anti-Nogo-A antibody-treated monkeys: *r*=0.191 for the vertical slots and *r*=0.162 for the horizontal slots (*P* = 0.682 and *P* = 0.729, respectively; red symbols in Fig. 4A and B). In other words, the anti-Nogo-A antibody-treated monkeys exhibited a contact time recovery mostly independent of the lesion extent. The data were processed statistically as described above (Fig. 2E and F). Contact time for the anti-Nogo-A antibody-treated monkeys was statistically significantly different from that of the control antibody-treated monkeys for both the vertical slots (Mann–Whitney: *P* = 0.008; Fig. 4C) and the horizontal slots (*P* = 0.005; Fig. 4D; see also Table 2, row A). Improvements in contact time were thus significantly better in the anti-Nogo-A antibody-treated monkeys than in the control antibody-treated monkeys using a statistical approach that considered the lesion extent.

As outlined in Materials and Methods (second statistical test), the overall functional recovery was assessed using a trivariate rank test on recovery of score, recovery of contact time and lesion extent, revealing a statistically significant difference between the anti-Nogo-A antibody-treated monkeys and control antibody-treated monkeys for the horizontal slots (*P* = 0.044) but not for the vertical slots (*P* = 0.072); see also Table 2, row B.

As compared to our recent report (Freund *et al.*, 2006a), one additional monkey was introduced here (Mk-AK); in this one the anti-Nogo-A antibody was not infused until 1 week post-lesion. This monkey exhibited complete recovery of function, as assessed by both the score (Fig. 2C and D) and the contact time (Fig. 4A and B), similar to what was observed in most anti-Nogo-A antibody-treated monkeys that received immediate post-lesion treatment.

### Re-examination of the behavioural data as a function of the volume of the cervical lesion

In our previous reports (Freund *et al.*, 2006a, 2007) and for data presented above, lesion extent was evaluated on the basis of the surface of the territory of injury expressed as a percentage of the corresponding hemi-cord surface, as seen on a 2-D reconstruction of the lesion in a single transverse plane of the cervical cord (Fig. 1B and C). Although the plane of reconstruction was taken at the level of maximal lesion extent in the transverse plane of the spinal cord, the percentage values of lesion extent as exhibited in Figs 2 and 4 did not account for the size of the lesion along other axes. Moreover, our previous report (Freund *et al.*, 2007) provided evidence that a part of the anti-Nogo-A antibody-enhanced density of CS axon arbors that were found caudal to the lesion had arisen from sprouting of the CS tract rostral to the lesion, and that had possibly migrated around the lesion site (for instance in the gray matter left intact by the injury). Thus, the expression of the lesion extent as a percentage of hemi-cord surface, as derived from a 2-D representation of the lesion as in Fig. 1B and C, may not fully account for the distance covered by regenerating axons. Therefore, as a complementary approach, the present study includes a 3-D analysis of the cervical lesion in order to present the estimated volume of the lesion, derived from an extrapolation on consecutive histological sections (see Materials and methods). Across monkeys, the estimated volume of the cervical lesion ranged from 1.348 to 6.961 mm^3^ (Table 1). In the control antibody-treated monkeys, the mean ± SD estimated volume of the lesion was 2.556 ± 0.77 and the median value 2.53 mm^3^ whereas, in the anti-Nogo-A antibody-treated monkeys, the mean estimated volume was 3.478 ± 2.01 and the median value 3.08 mm^3^. The estimated volume of the cervical lesion was thus somewhat larger in the group of anti-Nogo-A antibody-treated monkeys than in the group of control antibody-treated monkeys, but this difference was not statistically significant (Mann–Whitney test, *P* > 0.05). Similarly, the trivariate statistical approach applied to the percentage lesion extent, the volume of scar and the volume of penumbra did not show a statistically significant difference between the control antibody-treated monkeys and the anti-Nogo-A antibody-treated monkeys (*P* = 0.84).

Recovery of score (number of pellets retrieved in 30 s) was plotted as a function of the estimated volume of the lesion, for the vertical and horizontal slots, respectively, in Fig. 5A and B. Similarly, recovery of contact time was plotted as a function of the estimated volume of the lesion in Fig. 5C and D, for the vertical and horizontal slots, respectively. Functional recovery (both for the score and the contact time) was more prominent in anti-Nogo-A antibody-treated monkeys (largely irrespective of the estimated volume of the lesion) than in control antibody-treated monkeys (Fig. 5). In the latter group, the functional recovery for the score was inversely correlated with the estimated volume of the lesion for both the vertical slots (*r* = −0.937; *P* < 0.006) and the horizontal slots (*r* = −0.872; *P* < 0.024). Still in the group of control antibody-treated monkeys, the functional recovery for the contact time was inversely correlated with the estimated volume of the lesion in the horizontal slots (*r* = −0.899; *P* = 0.015) whereas this was only a trend in the vertical slots (*r* = −0.606; *P* = 0.202). In the anti-Nogo-A antibody-treated monkeys (Fig. 5), recovery was not correlated with the estimated volume of the lesion, as indicated by coefficients of correlation of *r*=0.026, *r*=0.097, *r*=0.293 and *r* = −0.148 in panels A, B, C and D, respectively, of Fig. 5; these were not statistically significant (*P* > 0.5).

To compare the recovery between the two groups of monkeys, the same statistical analysis was conducted on the plots of Fig. 5A–D as previously done in Figs 2E and F, and 4C and D. The results of the bivariate statistical analysis (functional recovery of either score or contact time as a function of the estimated volume of the lesion) are given in Table 2, row C. Considering both score and contact time, the statistical analysis confirms the notion of a significant enhancement of functional recovery in the anti-Nogo-A antibody-treated monkeys as compared to the control antibody-treated monkeys, when grasping was tested in both the vertical slots and the horizontal slots (see *P*-values in Table 2, row C). The trivariate analysis conducted on the parameters score, contact time and estimated volume of the lesion (Table 2, row D) showed that the difference between the two groups of monkeys was statistically significant for the horizontal slots (*P* = 0.031) but not for the vertical slots (*P* = 0.146).

### Specific behavioral and tract-tracing data derived from monkey Mk-AK treated with anti-Nogo-A antibody after a delay of 1 week post-lesion

In our previous report (Freund *et al.*, 2007), tract-tracing data were reported from nine monkeys subjected to cervical lesion. Behavioral data on the modified Brinkman board had been presented for eight of these animals (Freund *et al.*, 2006a). Four additional monkeys considered in Freund *et al.* (2006a) were not suitable for tract-tracing analysis of the CS tract. As mentioned above, the present study includes a new monkey (Mk-AK) subjected to a cervical lesion and treated with anti-Nogo-A antibody, but the onset of antibody administration was delayed by 1 week (Fig. 1A). For completeness of data presentation regarding this particular monkey Mk-AK, contact time data, the most pertinent manual dexterity parameter, are presented in Fig. S1A. Post-lesion, Mk-AK shows a 100% recovery of contact time. The reconstruction of the density of BDA-labeled CS axons rostral and caudal to the cervical lesion is shown in Fig. S1B. From such reconstructions, the axonal swellings caudal to the lesion were counted and normalized according to the total number of CS axons labeled at C5 level, as done for the animals in our previous reports (Freund *et al.*, 2006a, 2007). Furthermore, the CS axons crossing midline at the level of C5 were also counted and normalized as above. These two parameters for Mk-AK (normalized number of axonal swellings caudal to the lesion and normalized number of CS axons crossing midline at C5) are plotted in Fig. S1C and D as a function of lesion extent, together with the same data reported earlier for eight other monkeys (Freund *et al.*, 2007). For both parameters, data from Mk-AK aligned with the cluster of data points formed by the other anti-Nogo-A antibody-treated monkeys (red squares), largely separated from the cluster of data points formed by the control antibody-treated monkeys (blue circles).

## Discussion

The present study re-examines the behavioral recovery of 13 adult macaque monkeys with unilateral transection of the cervical spinal cord and subsequent infusion of an anti-Nogo-A or a control antibody. Using additional behavioral criteria and more detailed statistical methods, we confirmed our initial observations (Freund *et al.*, 2006a) clearly showing that anti-Nogo-A antibody treatment promotes functional recovery from cervical hemisection.

The present study adds significant information above and beyond our first report (Freund *et al.*, 2006a). First, our initial statistical comparison of score (number of pellets retrieved within 30 s, which represents a first outcome measure from the manual dexterity test) between the two groups of monkeys was univariate, taking into account only the score without considering the variability of the extent of the lesion (Freund *et al.*, 2006a). Whereas such a univariate statistical approach did not impact much on the group of anti-Nogo-A antibody-treated monkeys (in which the extent of recovery was largely independent of the size of the lesion), this is not true for the group of control antibody-treated monkeys, where the extent of recovery tends to be inversely correlated to the lesion extent (Figs 2C and D, 4A and B, and 5A–D). The bivariate statistical analysis (Figs 2E and F, and 4C and D, and Table 2, rows A and C, representing a secondary outcome measure from this behavioral investigation) is thus more appropriate, as it integrates the two variables. Second, the most original and valuable contribution of the present study is the introduction of the parameter contact time, which represents a very sensitive and robust measure of hand and finger dexterity (contact time is also a secondary outcome from the modified Brinkman board task). Indeed, considering the score data as a function of lesion extent (% of hemi-cord) in the bivariate analysis, the difference between the two groups of monkeys was significant at a *P*-value of 0.038 when the vertical and horizontal slots were cumulated (Freund *et al.*, 2006a) or at a *P*-value of 0.05 and 0.035 for the vertical slots and horizontal slots, respectively (Fig. 2E and F; Table 2, row A). Contact time data yielded a more significant difference between the control antibody- and the anti-Nogo-A antibody-treated monkeys, with a *P* = 0.008 for the vertical slots and *P* = 0.005 for the horizontal slots (Fig. 4C and D; Table 2, row A). Similarly, in the bivariate statistical analysis taking into account the estimated volume of the lesion (Table 2, row C), the difference between the two groups of monkeys was significant for both the contact time data and the score data for the two slot orientations (Table 2, row C). We believe that contact time data are more specific and robust than the score data because contact time reflects more specifically the function (finger dexterity) affected by the cervical lesion, whereas the score data are contaminated by other components of the overall behavior such as reaching, retrieval, food release to the mouth, chewing, etc. As shown earlier, the anti-Nogo-A antibody-enhanced recovery was paralleled by an increase in corticospinal axonal sprouting both caudal and rostral to the lesion (Freund *et al.*, 2006a, 2007; see also Fig. S1); it is possible that enhanced sprouting in other descending tracts may also play a role.

Regarding the possible role played by descending tracts other than the CS tract, it is important to emphasize that, in our studies (Freund *et al.*, 2006a, 2007; present study), only the CS tract was labeled with BDA. As a consequence, quantitative data were available only for the CS tract (see e.g. Beaud *et al.*, 2008). The question of the possible contribution of the other descending tracts can thus be addressed only indirectly. The lesion extent as seen on a transverse plane of the cervical cord can be subdivided into different sectors of the white matter (Table 1), corresponding to the dorsal column (containing the ascending sensory inputs of the dorsal column system; see below), the lateral column (containing the main portion of the CS and rubrospinal tract) and the ventral column (containing the tectospinal, reticulospinal and vestibulospinal tracts; see Lemon, 2008 for a review on the location of the descending tracts in the monkey). Although the number of animals in the present study is substantial for a nonhuman primate study, it remains, unfortunately, insufficient to address the individual role played by the various descending tracts in the recovery of function. First, the functional roles of the various descending tracts are not fully independent (Lemon, 2008). Second, it would be necessary to have several animals exhibiting similar CS tract lesions but different lesions of the other descending tracts, a condition that was not met in the present data. Tentatively, in the control antibody-treated monkeys, the two animals exhibiting the least spontaneous recovery had lesions with large estimated volumes (Table 1 and Fig. 5) and were thus possibly subjected to a lesion that affected more of the ventral column, the dorsal column and the rubrospinal tract than in the other monkeys (Fig. 1B). In contrast, in the anti-Nogo-A antibody-treated animals, the monkeys Mk-AF and, to a lesser extent, Mk-AM, showing less prominent functional recovery than the other five monkeys, did not exhibit lesions that specifically affected descending tracts other than the CS tract. In conclusion, the descending tracts other than the CS tract are likely to play a role in functional recovery, at least in control antibody-treated monkeys, but additional monkeys with a wider range of lesion properties will be needed to address this issue in more detail.

An intrinsic limitation of such lesion studies is the considerable variability between individuals, the impact being even more substantial given an animal model (monkeys) for which ethical considerations restrict the number of available animals. In rodents, the inter-individual variability is dealt with in part by increasing the number of animals. In the present study, inter-individual variability is due to the inevitable variations in location and extent of the lesion, as well as to parameters related to monkeys’ motivational and attentional states. As stated above, the variability related to lesion extent was taken into account here statistically, in contrast to our previous report (Freund *et al.*, 2006a), by introducing the bivariate, nonparametric statistical test assessment of both the percentage of recovery and lesion size. The statistical analysis was extended by introducing a trivariate statistical test, representing an overall degree of recovery assessed with two different behavioral measures, and integrating the impact of the lesion extent expressed as a percentage of the hemi-cord. The results of the trivariate approach confirms the observation of enhanced functional recovery of manual dexterity in anti-Nogo-A antibody-treated monkeys when retrieving pellets from the horizontal slots but not from the vertical slots (Table 2, row B; *P* = 0.072 for vertical slots and *P* = 0.044 for the horizontal slots). In the trivariate statistical analysis, when considering the lesion size assessed as the estimated volume of the lesion and the same two behavioral measures (Table 2, row D), the difference between the two groups of monkeys was significant for the horizontal slots (*P* = 0.031) but not for the vertical slots (*P* = 0.146).

As compared to our previous report (Freund *et al.*, 2006a), in which the score data in the modified Brinkman board task were cumulated for the vertically and horizontally oriented slots, the present study provides a separate description for the two slot orientations (Fig. 1E), with respect to both the score data (Figs 2 and 5; Table 2) and the contact time data (Figs 3–5 and Table 2). For both parameters (score and contact time), in the anti-Nogo-A antibody-treated monkeys (red squares in Figs 2C and D, 4A and B and 5), the post-lesion recovery was generally similar for vertically and horizontally oriented slots, close to 90–100% (except Mk-AF for vertical score, Mk-AF and Mk-AM for horizontal scores; Mk-AF and Mk-AM for vertical contact times and Mk-AF for horizontal contact time). The effect of the anti-Nogo-A antibody treatment was somewhat more prominent for the horizontal slots than for the vertical slots, as suggested by the bivariate and the trivariate statistical analyses where lower *P*-values were obtained when comparing the two groups of monkeys for the horizontal slots than for the vertical slots (Table 2). Along this line, the two control antibody-treated monkeys with the longest contact times for the horizontal slots (Fig. 3D) had the most damage to the lateral part of the dorsal column (Fig. 1C). This observation is consistent with the notion that sensory loss disrupts precision grip (Gilman & Denny-Brown, 1966; Johansson & Westling, 1984; Glendinning *et al.*, 1992;Leonard *et al.*, 1992). In contrast, the two anti-Nogo-A antibody-treated monkeys with complete CS tract lesions as well as damage to the lateral part of the dorsal column (Mk-AM and Mk-AP; Fig. 1C) did not show such an increase in contact time variability post-lesion for the horizontal slots (Fig. 3D). The anti-Nogo-A antibody treatment may thus also have reduced the impact of sensory deficits on precision grip and not merely acted on the motor components of the manual dexterity depending either on the CS tract or on other descending tracts.

As compared to the report of Freund *et al.* (2006a), a thirteenth anti-Nogo-A antibody-treated monkey (Mk-AK) was included here. This animal differed from the other six anti-Nogo-A antibody-treated monkeys in an onset of anti-Nogo-A antibody infusion delayed by 1 week. The goal of this manipulation was to test the impact of such a delay, which may be required in the case of spinal cord-injured patients in order to allow their stabilization and precise diagnosis before any intervention. These preliminary data in a single monkey suggest that a delay of 1 week does not negatively impact the anti-Nogo-A antibody-enhanced recovery, although confirmation on a larger number of animals with delayed infusion is needed. Concerning the monkey Mk-AK, one may argue that this animal exhibited good functional recovery because it was not affected by the early (i.e. during the first week) infusion of any fluid around the lesion in contrast to the other 12 monkeys, which may be detrimental to the recovery. This argument can be ruled out as the monkey Mk-AK received a perfusion of saline during the first week before administration of the anti-Nogo-A antibody from week 2 to week 5 post-lesion (see Fig. 1A). For this reason, inclusion of Mk-AK in anti-Nogo-A antibody treatment group is justified. As far as the functional recovery is concerned, delaying infusion of anti-Nogo-A antibody by 1 week could make recovery more difficult, as compared to animals treated immediately. However, the optimal time window for efficient anti-Nogo-A antibody treatment in monkeys remains to be determined.

In this report, we present new behavioral measures of manual dexterity (i.e. the contact time in the modified Brinkman board task) coupled with new statistical analyses, constituting a sensitive and robust assessment of functional recovery after cervical lesion in a nonhuman primate model of spinal cord injury, in spite of the inevitable inter-individual variability related to lesion characteristics. As illustrated in Table 1, the lesion extent expressed as a percentage of the corresponding hemi-cord seen in the transverse plane is similar for the two groups of monkeys, as is the percentage of the dorsal column, lateral column and ventral column injured. Furthermore, considering that our newly introduced estimates of lesion volume show no significant differences between treatment groups, the enhanced functional recovery in the anti-Nogo-A antibody-treated monkeys clearly cannot be explained by less extensive lesions in this group of monkeys as compared to those in the control antibody-treated group. Although the lesion size, derived from hemi-cord cross-sections (Fig. 1B and C) and expressed as extent of the lesion given as the percentage of hemi-cord affected, is limited to one axis it is nevertheless useful to assess which descending and ascending tracts have been affected by the lesion. To take into account the size of the lesion overall, the newly calculated lesion volume represents an important complementary assessment of lesions, in particular with respect to the possible distance that regenerating nerve fibers must traverse in order to establish new connections.

Finally, the new measure contact time (of the fingers with the first pellet) focuses on finger dexterity, a characteristic unique to primates (see Lemon & Griffiths, 2005; Lemon, 2008 for review), by assessing the retrieval time itself without taking into account the less controlled inter-pellet time interval. The inter-individual variability mainly due to lesion properties is in line with the strong variability also encountered in spinal cord-injured patients, thus making the present behavioral model in monkeys clinically highly relevant. In spite of this inter-individual variability, the behavioral measure of contact time and the multivariate statistical assessments considering the lesion extent in volume clearly demonstrate that anti-Nogo-A antibody treatment indeed promoted functional recovery from spinal cord lesion in primates. The anti-Nogo-A antibody treatment thus represents a promising approach for improved clinical intervention in spinal cord-injured patients, most probably in combination with complementary strategies.

## Abbreviations

BDA: biotinylated dextran amine
CS: corticospinal

## Supporting information

Fig. S1. Additional data from monkey Mk-AK. Panel A.
