# Supplementary material for: Anti-Nogo-A antibody treatment promotes recovery of manual dexterity after unilateral cervical lesion in adult primates – re-examination and extension of behavioral data
Source: Eur J Neurosci. 2009 Mar;29(5):983–96. doi: 10.1111/j.1460-9568.2009.06642.x (PMC2695186; doi:10.1111/j.1460-9568.2009.06642.x)
Supplement: Supplementary file 1 [file ejn0029-0983-SD1.doc]

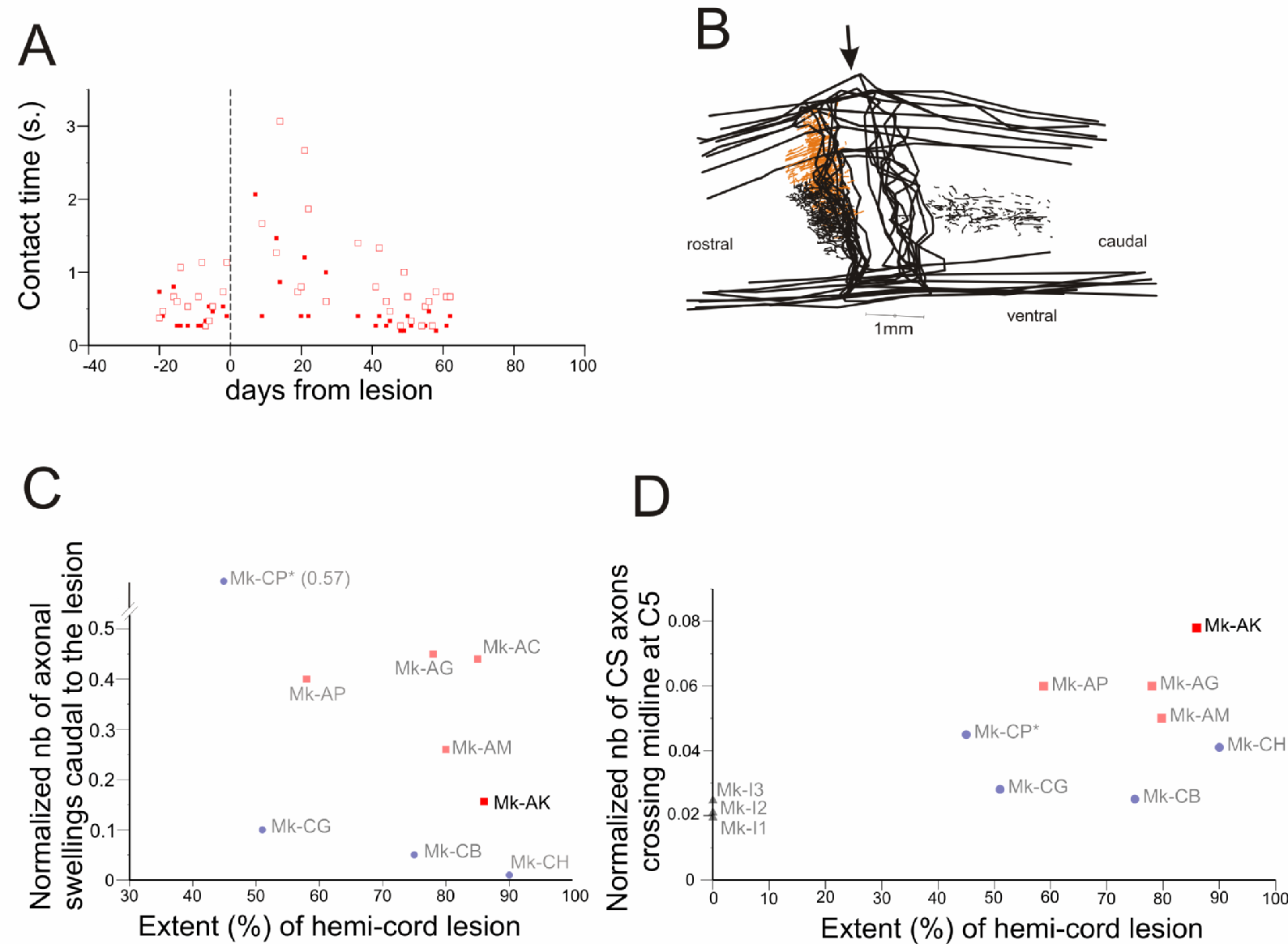


**Fig. S1**. **Additional data from monkey Mk-AK**.

Panel A: The “contact time” (in seconds), i.e. the time needed for one successful picking using the opposition of index finger and thumb in a pad to pad fashion, was plotted for the monkey Mk-AK as a function of time (days) with respect to the lesion. The monkey Mk-AK received the anti-Nogo-A antibody with an onset delayed by one week. Data are shown before the lesion and over the post-lesion weeks for the vertically (filled symbols) and horizontally (open symbols) oriented slots. On the abscissa, day zero (vertical dashed line) is for the time point of the cervical lesion. See Figure 1B for a representation of the cervical cord lesion.

Panel B: Superimposed reconstructions of sagittal sections of the cervical-thoraxic cord showing the lesion (arrow) and the density of BDA-labeled CS axonal arbors around the lesion in Mk-AK. Rostral to the lesion, the densely packed brown line segments represent the BDA-labeled CS axons in the white matter, as a result of BDA injection in the contralateral primary motor cortex, and interrupted by the lesion. The black line segments represent the CS axonal arbors rostral and caudal to the lesion in the gray matter. In this monkey, the dorsolateral funiculus was completely transected.

Panel C: For monkey Mk-AK (red symbol), the normalized number of CS axon swellings (*boutons terminaux* and *en passant*) was plotted as a function of the lesion extent, expressed in percent of the hemi-cord section. As a reminder, the same data obtained from the 8 monkeys reported in Freund *et al.* (2007) were represented, in pink for the anti-Nogo-A antibody treated monkeys and in light blue for the control antibody treated monkeys.

Panel D: For monkey Mk-AK (red symbol), the normalized number of CS axon collaterals crossing midline at C5 level was plotted as a function of the lesion extent, expressed in percent of the hemi-cord section. As a reminder, the same data obtained from the 7 monkeys reported in Freund *et al.* (2007) were represented, in pink for the anti-Nogo-A antibody treated monkeys and in light blue for the control antibody treated monkeys. The three black triangles along the ordinate are the same data but derived from intact monkeys (also derived from Freund *et al.*, 2007).

In panels C and D, the normalization was computed by dividing the number of axonal swellings (panel C) and of CS axon collaterals crossing midline (panel D) by the total number of CS axons found in the white matter at C5 level.
